# Metacognitive awareness and confidence as predictors of academic performance in pharmacy students: insights from grade predictions and structural equation modeling

**DOI:** 10.3389/fpsyg.2026.1720303

**Published:** 2026-02-27

**Authors:** Rihaf Alfaraj, Burhanettin Ozdemir, Lobna Aljuffali, Jawza F. Alsabhan, Noha Al Aloola, Hadeel Alkofide, Rana Aljadeed, Raniah Aljadeed, Faten Alodaib, Nora Alkhudair, Haya M. Almalag, Ghada A. Bawazeer, Lamya S. Alnaim, Njoud Altuwaijri

**Affiliations:** 1 Department of Pharmaceutics, College of Pharmacy, King Saud University, Riyadh, Saudi Arabia; 2 Department of Operations and Project Management, College of Business, Alfaisal University, Riyadh, Saudi Arabia; 3 Department of Clinical Pharmacy, College of Pharmacy, King Saud University, Riyadh, Saudi Arabia; 4 Department of Pharmaceutical Chemistry, College of Pharmacy, King Saud University, Riyadh, Saudi Arabia

**Keywords:** academic achievements, grade predictions, metacognition, performance, pharmaceutical education, student confidence

## Abstract

**Background:**

Pharmacy students often struggle with accurate self-assessment of learning outcomes. Many students overestimate exam performance, reflecting poor metacognitive awareness and overconfidence. This study examined the relationships between metacognitive awareness, self-confidence in grade predictions, and academic performance in pharmacy education, thus addressing how these factors interact without inferring causation.

**Methods:**

Pharmacy students (*n* = 151) at King Saud University participated in this study while enrolled in a Pathophysiology, Drug Action, and Therapeutics course. Surveys were pre-tested and post-tested twice at two midterm examinations to determine their self-reported metacognitive awareness and confidence. Students made predictions for future course grades. These self-tests were then contrasted to actual exam scores. Statistical analysis was performed using R software version 4.3.1, and students were categorized by metacognitive and confidence ability.

**Results:**

Despite underperforming on the first exam, students’ confidence in their grade predictions remained high. Students with better metacognitive awareness and well-calibrated confidence scored significantly higher on exams (*p* < 0.01). Metacognitive cognition and confidence were positively associated with academic performance (*r* = 0.467 and 0.361, *p* < 0.01), and with each other (*r* = 0.251, *p* < 0.01). Gender differences showed higher overall confidence and metacognitive cognition scores in males.

**Discussion:**

The results indicate that metacognition and confidence are critical for academic performance. These findings suggest that educational programs targeting self-evaluation warrant further investigation.

## Introduction

In an era of rapid knowledge expansion and technological change, the ability to *“think about thinking”—*known as metacognition*—*is increasingly recognized as a critical skill for effective learning and professional practice (Pate et al., 2019). Globally, metacognition is increasingly emphasized in health professions education as a core skill supporting self-regulation, adaptability, and lifelong learning.

Metacognition encompasses both awareness of one’s cognitive processes and the capacity to regulate them in pursuit of learning goals (Schraw, 2009). In other words, it involves knowing one’s strengths and limitations as a learner and being able to plan, monitor, and adjust one’s strategies during problem-solving or study (Ding et al., 2025). By enabling individuals to self-assess their understanding and adapt their approach in real time, strong metacognitive skills foster lifelong learning and adaptability; these qualities that are vital for future health professionals (Boldt and Gilbert, 2022).

Given the cognitive complexity and patient-safety implications inherent in pharmacy practice, especially for pharmacy students, well-developed metacognitive skills underpin critical thinking, self-directed learning, and clinical reasoning in patient care (Walker et al., 2023). The Accreditation Council for Pharmacy Education (ACPE) identifies self-awareness as a core domain calling on pharmacy programs to ensure that students can assess their learning and practice gaps as part of their professional development (ACPE, 2016). In Saudi Arabia, the National Center for Academic Accreditation and Evaluation (NCAAA) likewise mandates self-reflection and self-assessment skills in pharmacy education, thus identifying self-awareness and reflective learning as key learning outcomes for PharmD graduates (NCAAA, 2023).

The ability to reflect on one’s own thought processes allows student pharmacists to identify knowledge gaps, regulate their learning, and make informed decisions during complex therapeutic problem-solving (Steuber et al., 2017). In professional practice, metacognitive skills are essential for pharmacists working in both hospital-based clinical settings and community pharmacies, where continuous self-monitoring, reflection on therapeutic decisions, and adaptation to patient-specific factors are critical for safe and effective care. Previous studies have demonstrated a significant positive relationship between metacognitive awareness and academic achievement: Students who exhibit higher metacognitive awareness often have higher academic performance and higher grade point averages (Parsons, 2019).

Research shows that higher-achieving students typically display greater self-awareness and metacognitive engagements, which can translate into better academic performance and more robust clinical decision-making. Conversely, several studies have shown that many pharmacy learners exhibit suboptimal metacognitive accuracy and struggle to assess their performance realistically (Rivers et al., 2020). For example, one study found that pharmacy students were generally overconfident in predicting their presentation scores with the lowest-performing students overestimating their abilities the most (Steuber et al., 2017). Such findings suggest that a significant proportion of students are not yet adept at “thinking about thinking”, i.e., they lack accurate monitoring of their knowledge and may not recognize deficiencies in time to correct them. This gap in metacognitive proficiency is an educational concern, because students who cannot accurately self-evaluate are at risk of poor preparation or decision-making errors in both academic and clinical settings (Moraitou and Metallidou, 2021).

Academic confidence is a related factor that interacts with metacognition in learning. Confident students are generally more likely to engage in academic tasks, persist through challenges, and use effective study strategies (Stankov et al., 2017). However, confidence must be well-calibrated to be beneficial. Overconfidence can lead learners to underestimate the need to prepare or review (resulting in surprisingly poor performance), whereas under-confident students may avoid challenging tasks or experience debilitating anxiety. Indeed, evidence suggests that both excessive over-confidence and insufficient confidence can be detrimental. One recent study noted that students who were either over-confident or under-confident tended to have lower achievement potential and more internal conflict than those with well-matched confidence and ability (Douglas et al., 2023). It is important to distinguish between the item-level confidence judgments assessed in this study and the broader construct of domain-level self-efficacy. Self-efficacy, as operationalized in Lo's (2023) framework, refers to a relatively stable belief in one’s overall capability to succeed in a given domain—a belief that is built over time through mastery experiences, perceived control, and supportive instructional environments. In contrast, the confidence ratings collected in our study represent more granular, task-specific judgments of certainty made at a particular moment in time. A plausible developmental pathway, consistent with Lo's (2023) “emotional bridge” framework, is that hybrid learning structures that enhance perceived control gradually build domain-level self-efficacy, which then provides a foundation for more accurate and well-calibrated metacognitive monitoring at the task level.

These findings underscore the importance of calibrating between confidence and competence. By exploring metacognition alongside confidence, we aimed to capture a fuller picture of how students self-evaluate and regulate their learning before and after the assessments. This dual focus allows us to examine whether students’ confidence aligns with their actual performance (i.e., whether they are well-calibrated) and how any misalignment might impact their grade predictions and academic success (Medina et al., 2017). Moreover, it is worth clarifying that metacognition, while related to the concept of reflective practice, is distinct in timing and scope. Metacognitive monitoring and control occur *in real time* during learning or problem-solving: Metacognition enables immediate adjustments in strategy. In contrast, reflective practice typically occurs *after* an event—one looks on performance retrospectively (Walker et al., 2023). Both are valuable in health education; however, real-time metacognitive awareness is especially critical for preventing errors and optimizing decisions in fast-paced clinical scenarios (Newsom et al., 2022).

Despite the recognized importance of metacognition, explicit metacognitive training is still emerging in some educational contexts. The relationship between metacognition, academic confidence, and student performance has received increasing attention in educational research because of its role in improving self-regulation, adaptability, and lifelong learning (Zavadskiy et al., 2025).

In the Middle East, and Saudi Arabia in particular, the prevailing educational culture has historically emphasized rote memorization and recall over reflective or self-regulated learning (Yusuff, 2015). Many students from this traditional background have had limited practice in monitoring their own understanding or employing higher-order learning strategies. Recent reforms in Saudi pharmacy education—notably the nationwide transition to a Doctor of Pharmacy (PharmD) curriculum—signal a shift toward more active and student-centered learning (Aljuffali et al., 2024). These reforms have begun to introduce pedagogical strategies such as self-reflection exercises, case-based learning, and peer assessment aimed at cultivating higher-order thinking and metacognitive skills. However, the explicit integration of metacognitive skills training into the curriculum remains limited. Most pharmacy programs in the region do not yet systematically teach students *how* to plan, monitor, and evaluate their learning processes. Therefore, addressing this gap is crucial. Studies indicate that encouraging pharmacy students to engage in metacognitive activities (e.g., through guided reflection and feedback) can improve their critical thinking, deepen their learning approach, and boost their confidence in clinical decision-making (Zarei Hajiabadi et al., 2023). By weaving metacognitive practice into coursework, educators can help students develop the self-awareness needed to identify biases or errors in their thinking (Güner and Erbay, 2021) and foster the habit of lifelong learning to keep up with professional advances in the profession (Shah et al., 2020).

Considering these issues, this study evaluated the interplay between metacognitive awareness, confidence, grade predictions, and academic performance among pharmacy students. We examined this interplay in the context of 3rd-year Pathophysiology, Drug Action and Therapeutics course focusing on students’ self-evaluations before and after assessments. Specifically, we measured each student’s general metacognitive awareness, their expected exam grades, their confidence levels prior to the two midterm exams, their grade predictions, and their confidence after receiving feedback on each exam. This design enabled us to assess *prediction accuracy* (calibration) at different points and to observe how students adjusted their self-evaluations post-exam. We then analyzed how these factors correlated with the students’ actual exam performance. Our goal was to determine whether students with higher metacognitive awareness and well-calibrated confidence achieved better outcomes and helped identify patterns such as over- or under-confidence (miscalibration) that might distinguish different groups of learners (for example, by performance level or gender). By combining a general measure of metacognitive awareness with specific pre- and post-exam confidence judgments, this study provides a nuanced view of pharmacy students’ self-monitoring behavior during exams. We discuss how these insights can inform educational strategies to strengthen metacognitive skills, improve students’ confidence calibration, and support greater academic and professional success in pharmacy education.

This study examined metacognitive awareness, student confidence, and prediction accuracy as independent variables, while academic performance is treated as the dependent variable. We propose that higher metacognitive awareness is associated with more accurate self-performance predictions and better-calibrated confidence, which in turn relate to higher academic performance. Unlike previous studies that typically examine metacognitive awareness or confidence in isolation, this study integrates a general measure of metacognitive awareness with confidence calibration assessed both before and after examinations. This combined approach allows for a more dynamic examination of how self-awareness and confidence interact over time to influence academic outcomes, thus representing a novel contribution to the literature. Additionally, these cognitive and confidence processes can be understood within a broader affective-motivational context. Recent work by Lo (2023) introduces the notion of an “emotional bridge” in higher education, arguing that in hybrid and flipped learning environments, the design and structure of instruction directly shape students’ emotional states, self-efficacy, and engagement. Drawing on control-value theory (Pekrun et al., 2011), Lo demonstrates that five environmental variables—quality of instruction, induction of values, autonomy, goal structures, and achievement feedback—shape the achievement emotions that students experience. In such settings, teacher scaffolding, autonomy-supportive design, and formative feedback collectively build perceived control and self-efficacy, which in turn influence how accurately students monitor their own learning and how well they perform. By situating our investigation of metacognitive awareness and confidence within this framework, we position our research questions as part of the broader “emotional bridge” that connects classroom design features to academic achievement. This perspective suggests that the metacognitive monitoring and confidence calibration we examine are not purely cognitive phenomena but are embedded within, and shaped by, the affective-motivational climate of the learning environment.

### Aim and objectives

This study examined the relationships between metacognitive awareness, academic confidence, prediction accuracy, and academic performance among 3rd-year pharmacy students enrolled in a Pathophysiology, Drug Action, and Therapeutics course. Building on a dual-timepoint design, the study explored how students’ self-evaluations—measured both before and after midterm assessments—interact with actual exam outcomes.

In this framework, metacognitive awareness, confidence levels, and grade prediction accuracy are treated as the independent variables, while academic performance (exam score) is the dependent variable. Metacognitive awareness was assessed using a validated self-report measure, whereas confidence and prediction accuracy were operationalized through pre- and post-exam grade estimates. The aim was to investigate whether students with greater metacognitive awareness and more calibrated confidence were more likely to perform well academically, and whether prediction accuracy improved after feedback.

The study also examined potential gender differences in metacognitive awareness and confidence, and their implications for instructional strategies. By capturing cognitive (awareness and monitoring) and affective (confidence and expectation) variables in parallel, the research contributes to a more integrated understanding of self-regulated learning in pharmacy education. Findings from this study will inform future curriculum design by highlighting the role of metacognitive training and confidence calibration in promoting academic success.

### Methodology

#### Data collection, research instruments, and study design

A within-person repeated-measures design involving pre- and post-exam predictions across two midterms design was used to capture students’ metacognitive awareness, confidence, and academic performance at a single assessment time point without inferring causality. The study was conducted at King Saud University among 151 undergraduate pharmacy students enrolled in the Pathophysiology, Drug Action, and Therapeutics, a 3rd year- course. All enrolled students were included: 77 male and 74 female participants.

The research instrument consisted of three components, each item was rated on a 5-point Likert scale ranging from 1 = No idea to 5 = Very confident. Higher scores indicated greater perceived metacognitive awareness or confidence. First, metacognitive awareness was conceptualized as students’ knowledge of, and ability to regulate, their own cognitive processes including planning, monitoring, and evaluation of learning. This construct was assessed using self-reported items grounded in established metacognitive theory as also studied by Kim (2025).

Second, student confidence was operationalized as students’ perceived certainty regarding their predicted academic performance. Confidence ratings reflect metacognitive monitoring and confidence calibration, which describe how accurately learners judge their own knowledge and performance. This operationalization aligns with prior research demonstrating that confidence judgments provide meaningful insight into metacognitive monitoring processes (Coskun, 2018).

Third, prediction accuracy was defined as the discrepancy between students’ predicted exam scores and their actual exam scores. Grade prediction accuracy was assessed using students’ numeric estimates of their expected exam score (0–100). This metric indicated metacognitive calibration and has been widely used in metacognitive research as an objective marker of self-assessment accuracy (Knight et al., 2022).

Data were collected using a structured questionnaire administered 1 day before each midterm examination (conducted in the first and second half of the semester). The questionnaire included pre- and post-exam grade prediction items, confidence rating items using Likert scales, and self-assessment questions related to metacognitive monitoring and awareness (Supplementary Table S1). The instrument was developed based on prior studies in health professions education (Curtis et al., 2013; Karaca et al., 2023; Tabibzadeh et al., 2020). The instrument was adapted to the pharmacy education context, reviewed by two faculty experts in educational assessment and cognitive psychology, and pilot-tested for clarity with a small group of students not included in the final analysis. After completion of the examinations, actual exam scores were obtained from official course records and compared with students’ predicted scores. In line with canonical metacognitive awareness frameworks, the instrument represents an adapted brief scale capturing core aspects of metacognitive knowledge and regulation rather than a full multidimensional inventory. Internal consistency indices and item-level loadings are reported to support score interpretation, while acknowledging the scale’s limited scope and the resulting constraints on construct generalization.

All analysis tools in R (version 4.3.1) were operationalized and used to ensure methodological transparency and replicability. Ordered categorical variables were coded as full response anchors of survey items, and composite scores (Total_Cog, Overall_Conf and Total_Resp) were calculated by summing the responses of items after establishing internal consistency by using Cronbach alpha. Prediction prompts were typed in the form of continuous numeric variable based on the perceived score of the students in the exam. The patterns of missing data were analyzed and multiple imputation was done using mice package (van Buuren and Groothuis-Oudshoorn, 2011) and the analyses carried out on imputed data sets to minimize bias and maximize the chances of statistical significance.

### Statistical analysis

R software (version 4.3.1; R Core Team, 2023) was used to analyze the data. All statistical procedures were chosen to answer the research questions regarding the relationships among metacognitive awareness, confidence, prediction accuracy, and academic performance. The total cognition score (Total_Cog), confidence score (Overall_Conf), and total responses at the item level (Total_Resp) exam score out of 100 (Marks_100) were computed to represent each participant’s overall metacognitive cognition, self-reported confidence, total engagement with the test items, and exam scores, respectively.

Cronbachs’s alpha was used to test the reliability of the internal consistency of the exam response items as well as the confidence (metacognitive awareness) survey. To test the underlying measurement structure of the exam response items and the scale of confidence, confirmatory factor analyses (CFA) were performed using one-factor models. The items in the survey were ordinal, and thus CFA models were estimated with robust estimators of ordinal data. Model fit was measured with a variety of indices such as the chi-square statistic, Comparative Fit Index (CFI), Tucker–Lewis Index (TLI), Root Mean Square Error of Approximation (RMSEA), and Standardized Root Mean Square Residual (SRMR).

The paired-sample *t*-test was used to evaluate the accuracy of prediction prior to and after the midterm examination to determine the influence of assessment experience on metacognitive calibration among the students. Multiple linear regression analysis tested predictors of academic performance with exam score (out of 100) being the dependent variable. Independent variables were predicted grades before and after the examination, general confidence and total score of cognitive performance. *R*^2^ and *F* statistics were used to assess model fit and explained the variance.

A structural equation model (SEM) was used to explore further the interrelationships between metacognitive awareness, confidence, prediction accuracy, and academic performance. The SEM could model the latent performance constructs and latent confidence constructs as well as their direct influence on academic performance and prediction accuracy. The same set of global fit indices used in the CFA models were used to evaluate model fit. Marks_100, Total_Cog, Overall_Conf, and Total_Resp represent participants’ exam score out of 100, overall cognition, confidence, and total item-level responses, respectively.

## Results

The results of the analysis were based on the data of the students of pharmacy, who passed a midterm test and a post-exam survey about confidence and metacognitive awareness. The performance in academics was indexed using the standardized exam score. Seven confidence items were used to measure metacognitive awareness and confidence and the response to the exam served as a measure of performance in the midterm assessment. Metacognitive calibration was measured by comparing predicted grades prior to the exam with predicted grades after the exam. Table 1 shows internal consistency reliability estimates (Cronbachs alpha) of the items in the exam response and the confidence (metacognitive awareness) scale.

**Table 1:** Internal consistency reliability of study measures.

Scale	Number of items	Cronbach’s *α*
Exam responses (Q1–Q7 Resp)	7	0.74
Confidence/metacognitive awareness (Q1–Q7 Conf)	7	0.90

The confidence scale had a high internal consistency meaning that it was very reliable in measuring the perceived confidence and metacognitive awareness of the students. The relatively lower reliability of the exam response items is expected because the items indicate different exam questions and not a homogenous psychological construct. Considering the above, the responses in examinations were used as a measure of performance as opposed to being a psychometric scale.

The results of one-factor confirmatory factor analysis of the confidence scale are presented in Table 2 with standardized factor loading, global model fit indices.

**Table 2:** Confirmatory factor analysis (CFA) results for confidence (one-factor models).

Model fit indices					
Construct	χ^2^ (df = 14)	*p*	CFI	TLI	RMSEA
Confidence/metacognitive awareness	53.41	<0.001	0.99	0.99	0.11

Standardized factor loadings	
Item	Confidence
Q1	0.76
Q2	0.82
Q3	0.87
Q4	0.91
Q5	0.84
Q6	0.78
Q7	0.80

The measurement structure of construct is supported by the one-factor CFA models provided. The confidence model exhibited good construct validity as the standardized factor loadings were high on all the items and the CFI and TLI values were found to be significantly high. The RMSEA was higher than traditional cutoffs, but high factor loading suggests that the one-factor structure is an appropriate measure of the confidence and metacognitive awareness of students. The high RMSEA could be explained by the ordinality of data and strong inter-item correlation.

Table 3 provides Pearson correlation coefficients, which depict the strength and direction of the relationship between the study variables in relation to Midterm 1. The variables that were used during the analysis were: gender, exam score (Score_100), total responses, total cognition, prediction before the exam (Pred_before), prediction after the exam (Pred_After) and overall confidence. The table has asterisks which show the level of statistical significance, which reflect the strength of the associations between the variables. This discussion gives an insight into the correlation between the cognitive engagement and prediction accuracy and confidence levels of students and academic performance.

**Table 3:** Pearson correlations among midterm variables.

Variable	Gender	Total scores (Marks_100)	Confidence score	Pred before	Pred After	Total responses	Cognition score
Gender	1	−0.124	−0.177*	0.001	−0.066	−0.188*	−0.224**
Total scores (Marks_100)		1	0.282**	0.225**	0.386**	0.835**	0.467**
Confidence score			1	0.016	0.316**	0.251**	0.361**
Pred_before				1	0.288**	0.110	0.161*
Pred_After					1	0.271**	0.415**
Total responses						1	0.410**
Cognition score							1

*p < 0.05, **p < 0.01.

The correlation analysis demonstrates that there are a number of statistically significant relationships between the study variables. Overall confidence, total responses and total cognition scores have a negative correlation with gender, but the correlation is weak. The total responses and total cognition scores, overall confidence, pre-exam predictions, and post-exam predictions all exhibit strong positive correlations with exam scores (Marks_100). The overall confidence is positively associated with the post-exam predictions, total responses and total cognition. The correlation between pre- and post-exam predictions is moderate which shows that there is consistency in the self-evaluation of students. The total response scores as well as total cognition scores have strong and moderate correlations with exam scores and post-exam predictions, respectively, and overall confidence, respectively.

The heatmap that represents Pearson correlation coefficients gives a relationship between academic and demographic variables in relation to Midterm 1 performance. The intensity of these associations and their direction is visually demonstrated by the color gradient; green-grey-red. Exam scores (Marks_100), total responses, and total cognition are strongly correlated with each other which is depicted by darker green shading. This implies that students whose scores were higher on the midterm also showed higher levels of cognitive and confidence. The relationship between gender and total responses and total cognition is weak and negative meaning that the scores of the students (male) who were coded as 1 had a slight difference in the variables. There are also moderate positive relationships observed between overall confidence, post-exam predictions, and exam scores, which indicate that the self-confidence of the students and post-exam assessment are moderately related to the actual performance. The heatmap highlights the relationship between cognitive, behavioural, and demographic factors that predict academic performance.

Table 4 represents the outcomes of independent samples *t*-tests of male and female students on major variables. It reports *t*-statistic, degrees of freedom, level of significance, difference between means, and 95-percent confidence intervals of each of the comparisons.

**Table 4:** Independent samples *t*-test for equality of means for midterm exam.

Variable	*t*	df	Sig. (2-tailed)	Mean difference	95% confidence interval
Total score (Marks_100)	1.520	149	0.131	2.95	(−0.89, 6.79)
Overall confidence	2.195	149	0.030	0.48	(0.05, 0.92)
Pred_before	−0.009	149	0.992	−0.01	(−1.10, 1.09)
Pred_After	0.803	149	0.423	0.35	(−0.51, 1.21)
Total responses	2.331	149	0.021	0.59	(0.09, 1.10)
Cognitive score	2.808	149	0.006	2.19	(0.65, 3.74)

Multi-group CFA was used to test measurement invariance between the genders. Configural and metric invariance were supported (*Δ*12 = 16.73, *p* = 0.16; ΔCFI = 0.003), which implies that there are no differences between the factor structure and loading between gender. It did not support scalar and latent mean invariance (Δ*χ*^2^ (14) = 36.57, *p* = 0.001; *Δ*2 (2) = 9.53, *p* = 0.009), which implies that there are differences in item intercepts and latent means. Thus, gender relationships comparisons are acceptable, whereas latent comparisons of means need to be applied with care.

Table 4 presents several statistically significant and non-significant findings regarding gender differences across the measured variables. Positive results indicate differences in favor of female group. For exam scores (Marks_100), male students have slightly higher mean scores than females; however, this difference is not statistically significant (*t*(149) = 1.520, *p* = 0.131). Statistically significant gender differences are observed in overall confidence, with males reporting higher confidence than females (*t*(149) = 2.195, *p* = 0.030). In terms of metacognitive ability, males also score significantly higher than females on the Total Cognition scale (*t*(149) = 2.808, *p* = 0.006). No statistically significant gender differences are found for grade predictions before (Pred_before) or after (Pred_After) the exam, nor for total response counts (Total_Resp). These findings indicate that while male and female students perform similarly on the exam, males tend to report greater metacognitive awareness and academic confidence.

Prediction bias (Predicted–Actual score) and absolute prediction error (|Predicted–Actual score) was used to operationalize prediction calibration with the lower absolute error representing higher calibration accuracy as shown in Table 4. An absolute prediction error before the examination and after was compared by a paired-samples t test. Findings showed no statistically significant difference between pre-exam and post-exam predictions based on absolute prediction error, *t*(150) = 0.90, *p* = 0.37, and 95 percent confidence interval of the mean difference [−0.35, 0.94]. The related effect size was inconsequential, Cohen *d z* = 0.07 and nature of change in calibration accuracy was trivial within persons (Table 5).

**Table 5:** Paired comparison of absolute prediction error before and after the examination.

Measure	Mean difference (post−pre)	*t*(df)	*p*	95% CI for mean difference	Cohen’s *d*
Absolute prediction error	0.29	0.90 (150)	0.37	[−0.35, 0.94]	0.07

These results show that though the students changed their grade predictions once they had taken the exam, this change could not be converted to higher accuracy in calibration in terms of absolute prediction error. In this regard, all interpretations in the manuscript have been reformulated to differentiate between variations in the level of prediction and variations in calibration, and assertions of the existence of better prediction accuracy are now limited to well-calculated calibration indices.

Table 6 presents the results of a multiple linear regression analysis predicting academic performance (exam score out of 100) from predicted grades, overall confidence, and total cognitive performance.

**Table 6:** Multiple regression predicting academic performance (Marks_100).

Predictor	*B*	SE	*β*	*p*
Predicted grade (before exam)	1.63	0.41	0.34	<0.001
Predicted grade (after exam)	0.12	0.38	0.03	0.75
Overall confidence	−2.02	1.07	−0.16	0.062
Total cognitive score	0.40	0.17	0.19	0.018

Model statistics: *R*^2^ = 0.299, *F*(4, 146) = 12.34, *p* < 0.001.

The regression model explained approximately 30% of the variance in academic performance. Prediction accuracy before the exam emerged as the strongest predictor of exam scores, highlighting the importance of accurate pre-assessment self-evaluation. Cognitive performance also contributed positively to achievement. Confidence showed a negative trend that did not reach statistical significance, indicating that higher confidence alone did not translate into improved academic outcomes.

Table 7 reports the standardized path coefficients and model fit indices for the structural equation model examining relationships among metacognitive awareness, confidence, prediction accuracy, and academic performance.

**Table 7:** Structural equation model of metacognition, confidence, and academic performance.

Path	Std. estimate (std. lv)	*p*
Cognitive performance → confidence	0.88	<0.001
Exam performance → Marks_100	0.91	0.002
Confidence → prediction accuracy	−0.21	0.19
Confidence → Marks_100	−0.15	0.27

Model fit: *χ*^2^ (115) = 231.92, robust CFI = 0.93, robust TLI = 0.92, robust RMSEA = 0.07, SRMR = 0.09.

Estimation of the structural equation model was done through robust maximization likelihood (MLR) to address the non-normality and mixed measurement levels. It was found that the model had acceptable to good fit considering the strong indices (Robust CFI = 0.93, Robust TLI = 0.92, Robust RMSEA = 0.07), which were used to verify the sufficiency of the given structural relations. The results with using standardized latent-variable estimates showed that cognitive performance was a strong positive predictor of confidence (0.88, *p* <0.001) and that greater metacognitive awareness and monitoring were connected to greater self-reported confidence. Academic outcomes were also associated with exam performance (0.91, *p* = 0.002).

Confidence, on the contrary, was not a significant predictor of prediction accuracy (−0.21, *p* = 0.19) or final exam scores (−0.15, *p* = 0.27). Based on these findings it is apparent that there is no mediating influence of confidence between the metacognitive processes and academic performance. Rather, there seems to be a direct effect of metacognitive and performance-based cognitive processes, and the statistically independent effect of confidence on calibration accuracy and achievement when these are adjusted.

The diagram represents standardized path estimates in which cognitive performance has strong effects to confidence and exam performance on marks with non-significant effects of confidence to prediction accuracy and exam performance.

Overall, higher cognitive performance was associated with confidence among students, and academic outcomes were directly related to exam performance. Confidence was, however, not significantly associated with prediction accuracy or exam scores and so suggests that item-level self-confidence is indicative of metacognitive awareness, but not a predictor of performance on its own. These findings confirm the contribution of confidence calibration to metacognitive monitoring but emphasize that cognitive ability and real performance in exams are more important predictors of academic success.

## Discussion

This research examines the association between metacognitive awareness, confidence level, and grade expectations in 3rd-year PharmD students in King Saud University, as a part of a course in Pathophysiology, Drug Action and Therapeutics. The findings provide information on the perception of the pharmacy students about themselves and how their self-assessment, confidence, and academic performance interrelate at pre- and post-assessment periods. While our findings offer insights that may inform broader educational strategies, their generalizability to other pharmacy education contexts may be limited due to the specific institutional and curricular setting in which the study was conducted. Unlike many prior studies that examined metacognition or confidence in isolation, our study integrates metacognitive awareness with pre- and post-exam confidence calibration, offering a more dynamic perspective on students’ self-monitoring over time.

The current results can be most readily explained in existing paradigms of metacognitive calibration and awareness. The most popular metrics used as calibration measures to predict and actual performance are bias and absolute accuracy which is good evidence of metacognitive monitoring accuracy (Ackerman and Goldsmith, 2011; Dunlosky and Thiede, 2013). Studies have always found that highly calibrated learners display better self-regulated learning patterns and overall academic performance, especially within learning systems that are assessments-based (Bjork et al., 2013; Deslauriers et al., 2019). Simultaneously, confidence judgments and metacognitive awareness were demonstrated to be at the center of the capability of learners to assess, control, and modify their learning strategies, which supports the significance of these mechanisms as the key factors contributing to academic performance (Rivers et al., 2020).

Our findings align with trends observed in prior reports. For instance, Isaacs et al. (2022) demonstrated a significant correlation between metacognitive awareness, mastery goals, and academic achievement, thus highlighting the role of self-regulatory learning strategies in promoting academic success. This conclusion is consistent with our study, where students exhibiting higher metacognitive awareness, particularly those able to better calibrate their predicted and actual scores, were associated with higher grades.

These findings can be interpreted through the lens of mastery goal theory. Mastery goals (also known as *learning* goals) refer to an achievement orientation centered on developing competence, understanding, and personal improvement rather than on outperforming others. In a mastery-goal framework, students focus on learning the material and improving their skills, which contrasts with performance goals that emphasize demonstrating superior ability or avoiding looking incompetent in comparison to peers. Learners who adopt mastery goals tend to engage in adaptive learning behaviors, e.g., they seek out challenges, persist despite difficulties, and use deep learning strategies. This can lead to positive outcomes such as greater enjoyment of learning and improved mastery of content. In contrast, a performance-goal orientation often correlates with more surface-level strategies and avoidance of challenging tasks, which can impede deep learning (Keus and Haave, 2020). This aligns with prior research. For example, Coutinho (2007) found that students with a strong mastery goal orientation demonstrated greater use of metacognitive strategies. Importantly, both mastery goals and metacognitive awareness were significant positive predictors of academic success (e.g., a higher GPA) (Coutinho, 2007). This pattern is further supported by recent large-scale evidence showing a significant positive association between metacognition and academic achievement across educational levels (Xie et al., 2024).

Our findings similarly showed that students with higher metacognitive awareness—particularly those with more accurate grade predictions—achieved better exam outcomes. Other recent studies align with this pattern echoing recent evidence that mastery-oriented students tend to exhibit stronger metacognitive monitoring and better academic outcomes (Jo et al., 2021).

Although mastery goal orientation was not directly measured here, this framework does provide a useful lens for interpreting why students with higher metacognitive awareness demonstrated better calibration and academic performance.

Recent studies in higher education and health professions education corroborate the beneficial interplay of metacognition and mastery-oriented motivation on academic outcomes. For example, Siqueira et al. (2020) reported that among medical students, those endorsing a mastery-approach orientation (prioritizing skill development, deeper learning, and personal improvement) scored significantly higher on metacognitive awareness measures.

This finding reinforces the link between a learning-focused goal orientation and strong metacognitive skills, which collectively support better learning strategies and academic success. Similarly, a 2025 study of medical undergraduates found that students with greater metacognitive regulation abilities and higher intrinsic motivation (an indicator of mastery-driven motivation focused on internal growth) achieved better academic performance. In that study, metacognitive self-regulation was positively correlated with students’ intrinsic goal motivation and with their exam outcomes, thus underscoring how metacognitive awareness and a mastery goal mindset jointly contribute to academic achievement (Deshmukh et al., 2025).

Taken together, our results and prior studies underscore that metacognitive awareness and mastery-oriented motivation function synergistically to support effective learning and self-evaluation in pharmacy education. However, the relationship between metacognitive awareness and performance must also be interpreted in context. As Guskey (2007) noted, performance gaps do not always correspond to differences in student confidence (Guskey, 2007). Indeed, our data show that students’ confidence levels remained relatively stable, yet they were not consistently aligned with actual performance. This supports Breedt and Pieterse (2012) view that confidence is a multidimensional construct that is shaped by factors such as self-efficacy, prior feedback, and attributional style. In other words, high-performing students were not always the most confident, and highly confident students did not always perform well, this imbalance was also observed in previous metacognitive research by Hacker et al. (2008).

This mismatch between confidence and competence may also reflect psychological mechanisms such as the self-fulfilling prophecy—where expectations influence outcomes—and self-handicapping. The self-fulfilling prophecy, first described by Merton, suggests that expectations can influence outcomes. In education, teacher or student expectations can shape performance over time (Jussim and Harber, 2005). On the other hand, self-handicapping occurs when students undermine their own performance (e.g., through procrastination or reduced effort) to protect their self-image in case of failure (Urdan and Midgley, 2001). These mechanisms provide one possible explanation for why some students with high confidence did not perform as well as expected: Their confidence may have been buoyed by optimistic expectations rather than careful preparation potentially leading to under-preparation (a self-handicapping behavior). Conversely, students with low confidence might perform below their capability due to anxiety or low expectations (a self-fulfilling prophecy effect). Prior research supports these interpretations: Jussim and Harber (2005) noted that teacher expectations can become self-fulfilling prophecies in the classroom, and Kumar (2019) highlighted that students with a history of success or failure often develop self-handicapping strategies that impact performance. Together, these perspectives underscore that the relationship between confidence and performance is complex and mediated by individual mindsets and contextual expectations.

To measure metacognition and confidence, the researchers used various variables such as predictions of grades before and after the exam, mean scores on the confidence scale, the accuracy of confidence on a per-item basis and a metacognitive awareness scale (total cognition). The Pearson correlation analysis showed that there were strong positive relationships between academic performance and total responses and metacognitive awareness scores. There were moderate positive relationships between academic performance and confidence and accuracy of prediction.

The measures showed internal consistency: The Cronbach alpha of both confidence and performance scale showed more than 0.7. CFA endorsed the factorial structure, and most factor loadings were greater than 0.70 with acceptable model fit (CFI = 0.91, TLI = 0.90, RMSEA = 0.08, SRMR = 0.09), which demonstrated credible and valid measurement of metacognition and confidence (Brown, 2015; Kline, 2016).

Regression analyses revealed that cognitive performance was a strong predictor of confidence, and confidence was not a significant predictor of prediction accuracy or exam scores. The performance in exams was strongly associated with Marks_100, which indicates that real performance is more effective than self-reported confidence in metacognitive calibration (Fleming, 2024; Kleitman and Stankov, 2007; Tsingos-Lucas et al., 2016).

SEM assisted in the conceptual dichotomy of metacognition, confidence, and performance. Confidence was highly predicted by cognitive performance (1.04, *p* < 0.001), and Marks_100 was predicted by exam performance (1.02, *p* = 0.002). The non-significant confidence to prediction accuracy or Marks_100 pathways imply that affective confidence is not the primary factor that enhances performance; proper self-monitoring and metacognitive skill development should be considered (Bozgun and Akin-Kosterelioglu, 2023; Callender et al., 2016; Rouault et al., 2018).

This study used multiple indicators to measure students’ metacognition and confidence including pre- and post-exam performance predictions, confidence ratings (confidence scores) on their answers, and a metacognitive self-assessment scale. This approach aligns with recent work in health professions education that evaluates metacognitive accuracy by comparing students’ predicted performance to their actual scores. For example, Powell et al. (2021) asked pharmacy students to predict their quiz scores and calculated the bias between predicted and actual performance as a measure of metacognitive skill. Similarly, Martirosov and Moser (2021) had students estimate their examination outcomes and observed that calibration accuracy varied across performance groups. We also administered a cognition-focused metacognitive inventory (similar to a Metacognitive Awareness Inventory) to assess students’ self-knowledge and regulatory skills following recommendations to use such surveys for evaluating metacognitive awareness. In medical education, comparable confidence-calibration methods have been used during clinical simulations to compare students’ self-rated confidence with actual performance, thus revealing patterns of overestimation in novice learners (Garbayo et al., 2023).

Our findings correspond to the recent literature on pharmacy education. Tsingos-Lucas et al. (2017) revealed that reflective writing competence is an indicator of academic achievement, regardless of the type of assessment. Structured reflection activities enhance metacognitive development and learning control (Tsingos-Lucas et al., 2016). Rivers et al. (2020) highlighted the significance of monitoring and control in the performance of pharmacy students indicating that students who are more metacognitively regulated have more positive results.

According to Tsingos-Lucas et al. (2016), reflection mediates between theory and practice to enhance the level of learning. Similarly, Walker et al. (2023) affirmed that pharmacy students use metacognitive skills such as planning and monitoring in the process of making therapeutic decisions, which improves their problem-solving abilities.

Walker et al. further confirm that pharmacy students naturally employ metacognitive strategies like planning and monitoring during therapeutic decision-making. Doing this enhances their problem-solving abilities (Walker et al., 2023). Collectively, these studies provide a context for our findings: The high achievers in our course not only *knew* more (as evidenced by exam scores), but they also *thought* more about their own thinking, i.e., planning, monitoring, and evaluating their understanding. This in turn likely contributed to their success.

In practical terms, students who scored higher on the exam tended to have higher metacognitive awareness and provided more confident responses on the test, thus indicating greater cognitive engagement. This aligns with other recent findings: Kleitman and Stankov (2007) reported that self-confidence shares a strong relationship with metacognitive processes like monitoring and control of cognition.

In our study, gender emerged as a noteworthy factor in metacognitive self-assessment. Male students showed significantly higher mean scores in overall confidence and metacognitive cognition (knowledge/awareness) compared to female students (*p* < 0.05), although exam performance did not differ significantly between genders. No significant gender differences were observed in grade prediction accuracy (before or after the exam) suggesting that males and females were similarly calibrated in predicting scores, even if their confidence levels differed. These results indicate that while academic ability was comparable, male students reported greater confidence and self-rated metacognitive skill.

This pattern mirrors some prior findings in the literature. For instance, Zeegers (2004) noted that male undergraduates tended to report higher use of metacognitive strategies than their female counterparts, and Stankov and Lee (2008) observed that male students often express higher confidence (sometimes to the point of overconfidence) on academic tasks. These findings reflect prior gender patterns; however, evidence on confidence-per-item and metacognitive strategy use by gender is mixed. While earlier work noted males reporting higher strategy use, more recent studies find no inherent gender advantage in confidence or metacognitive ability (Xue et al., 2024). In pharmacy education, gender differences have also been observed in students’ learning preference profiles (e.g., VARK modalities), which may influence how male and female students engage with metacognitive strategies and self-monitoring during learning and assessment (Lee, 2025; Malik et al., 2021).

Such differences in learning preferences, combined with sociocultural expectations within professional programs, may contribute to variations in self-reported metacognitive strategy use rather than reflecting true differences in metacognitive ability (Agrawal et al., 2025). In certain contexts (e.g., teacher education), female students have even demonstrated higher metacognitive awareness than males (Ramadani et al., 2025). In our context, male participants had higher Total Cognition and confidence scores consistent with those trends. This pattern may reflect gender socialization and confidence-expression norms, where male students may be more likely to report higher self-assessed strategy use and confidence, while female students may provide more conservative self-ratings despite similar performance (Hofer et al., 2025; Reilly et al., 2022).

It is important to consider why these gender differences might exist. One explanation lies in sociocultural influences on learning behavior. Research in educational psychology suggests that gender socialization can shape students’ academic self-concept and confidence (Hofer et al., 2025). Males are often encouraged from an early age to project confidence in their abilities, which can lead to a higher self-reported confidence independent of actual performance (Stankov and Lee, 2008). In contrast, females may internalize more self-critical or modest attitudes about their competence potentially leading them to underestimate or under-report their capabilities even when performing well. For example, Pajares (2002) found that adolescent boys reported greater confidence in learning whereas girls were more engaged in self-reflective and evaluative learning tasks. This suggests that female students might be more cautious and critical in self-assessment—this is a double-edged sword that can promote deep reflection but also lower self-confidence. In our pharmacy education setting, such dynamics could mean that female students are equally capable but less likely to rate their own knowledge and skills as highly as male students do. This interpretation is bolstered by the lack of difference in actual exam achievement between genders, i.e., the divergence was in perception (confidence and self-assessed cognition) and not in performance outcomes. These sociocultural and pedagogical considerations collectively suggest that the observed gender differences in self-reported metacognitive awareness are best understood as context-dependent and perception-driven rather than as indicators of inherent ability differences.

The role of the educational environment and pedagogy is also important. The context of our study—a Pathophysiology and Therapeutics course—involves interactive learning and high-stakes assessments that might differently impact students based on gender. Some literature posits that classroom interactions and instructor expectations can create subtle biases; for instance, male students might receive (or perceive) more encouragement to speak up, take risks, or display confidence in class, whereas female students might be reinforced (or predisposed) to be careful and precise (Reilly et al., 2022). Over time, these experiences can amplify confidence differences. Additionally, there is the concept of stereotype threat in academic settings. We did not directly study these aspects, but they do provide a plausible backdrop for interpreting our results: Cultural and pedagogical factors likely intersect to produce the observed gender pattern in metacognitive self-evaluations.

Considering these points allows us to underscore how metacognitive awareness and confidence are intertwined with social context. The finding that males reported higher confidence and metacognitive awareness, despite similar achievement, suggests a need for educators to foster an environment where all students can accurately appraise and confidently express their abilities. This could involve incorporating metacognitive training and discussions about self-assessment that are mindful of gender-inclusive practices. By acknowledging and addressing these differences, faculty can help ensure that female students are not undervaluing their performance (and potentially missing opportunities for self-improvement or leadership), and that male students maintain confidence without becoming complacent or overconfident. Future research should explore these interventions and investigate whether our observed gender effects hold true in other institutions or cultural settings. If they do, then this reinforces the idea that the phenomena we observed are not isolated. Understanding the underlying causes, whether they be early educational experiences, societal expectations, or curriculum design, will be crucial for developing more personalized and equitable learning support.

**Figure 1:**
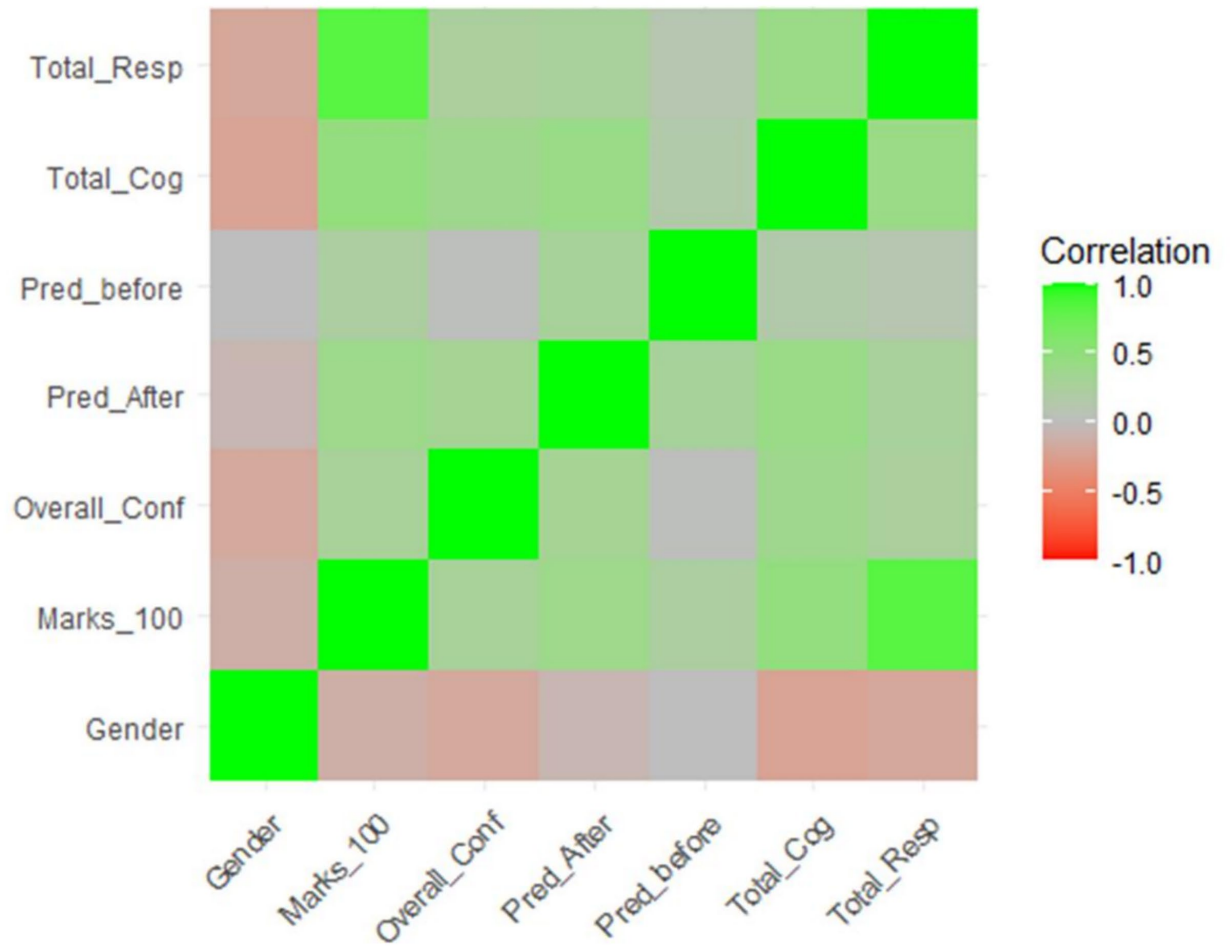
Heatmap of the variables.

Pearson correlation analysis revealed strong positive associations between academic performance and both total responses and metacognitive awareness scores. There were moderate correlations with confidence and prediction accuracy. Exam scores were strongly associated with cognitive engagement (Total Cognition) and the quantity of confident responses (Total Response) indicating that high-performing students tend to be both more confident and more cognitively engaged (Figure 1). The correlation seen between confidence and post-exam predictions aligns with the work of Kleitman and Stankov (2007) who found that confidence was associated with more accurate monitoring and control of cognition.

There was also increased prediction accuracy following the midterm and a significant mean change in the grade expectations of students. Nevertheless, there is a moderate positive relationship between pre- and post-exam predictions indicating that students could base their expectations after the test on their initial assumptions—this is in line with the anchoring effect as described by Hacker et al. (2000). Without alignment of initial confidence, the students might not be able to re-calibrate their expectations despite the feedback. Prediction accuracy also improved after the midterm. However, the moderate correlation between pre- and post-exam predictions suggests an anchoring effect; the initial beliefs unduly influence later judgments. This bias is well-documented in recent research. For example, students often use provided performance feedback as an anchor for subsequent confidence ratings (Urban and Urban, 2021). Beyond cognitive biases like anchoring, the emotional context of the learning environment can also influence students’ calibration. Lo (2023) cautions that an assessment-centric classroom climate can heighten anxiety and undermine self-efficacy, even when students are actively engaged. This perspective offers a valuable lens for interpreting our finding that calibration accuracy did not significantly improve from pre- to post-exam (Cohen’s *d* = 0.07): the high-stakes nature of the midterm examinations may have created an emotionally charged environment that limited students’ capacity for accurate self-assessment, despite the course’s use of flipped components and digital practice. Conversely, Lo (2023) demonstrates that autonomy-supportive design features—such as student-led learning, low-stakes formative tasks, and timely mastery-oriented feedback—are associated with more stable positive emotions and greater persistence, conditions that are likely to foster better metacognitive monitoring. In our course context, the Pathophysiology, Drug Action, and Therapeutics course incorporated some elements of active learning, yet the assessment ecology remained predominantly summative. This imbalance may explain why students’ confidence remained high but their calibration accuracy did not meaningfully improve: the emotional climate may not have been sufficiently supportive to translate engagement into genuine metacognitive growth. Integrating our findings with Lo's (2023) framework, we can propose a succinct interpretive pathway that connects classroom design to academic outcomes through affective and metacognitive mechanisms. In this model, the hybrid design of the course and the instructor’s pedagogical supports (e.g., pre-class materials, in-class active learning, formative assessments) shape students’ perceived control and achievement emotions. These affective-motivational factors, in turn, influence the development of domain-level self-efficacy. Higher self-efficacy then supports more effective metacognitive monitoring, as reflected in lower absolute prediction error and better-calibrated confidence judgments. Finally, improved monitoring contributes to stronger academic performance. Our data are consistent with this pathway: cognitive performance was a strong predictor of confidence (*β* = 0.88, *p* < 0.001), and exam performance was strongly associated with final scores (β = 0.91, *p* = 0.002), while confidence alone did not significantly predict calibration or achievement. This pattern suggests that the link between self-assessment and performance is mediated by deeper cognitive and affective processes rather than by surface-level confidence. Presenting this as a testable model positions our work for future confirmatory studies that could employ longitudinal designs and direct measures of self-efficacy and achievement emotions to validate these proposed relationships. Even arbitrary reference points can shift study behaviors—longer suggested times lead to longer study durations and better recall thus demonstrating an anchoring-and-adjustment in learning (Li et al., 2024).

The occurrence of this phenomenon creates a necessity in designing instructional interventions that enhance metacognitive calibration—especially among students who tend to be over-confident or tend to doubt themselves. The results of Callender et al. (2016), Lytra and Drigas (2021), Steuber et al. (2017), and Zabrucky and Bays (2011) confirm the necessity of developing more realistic self-assessment plans in educational institutions.

Post-exam confidence was not necessarily predictive of success. This finding is consistent with the results of Perry et al. (2018) and Snow et al. (2018) who established that students who hold inflated confidence tend to overestimate their abilities. These findings underscore the need to educate students to assess their knowledge and skills correctly.

**Figure 2:**
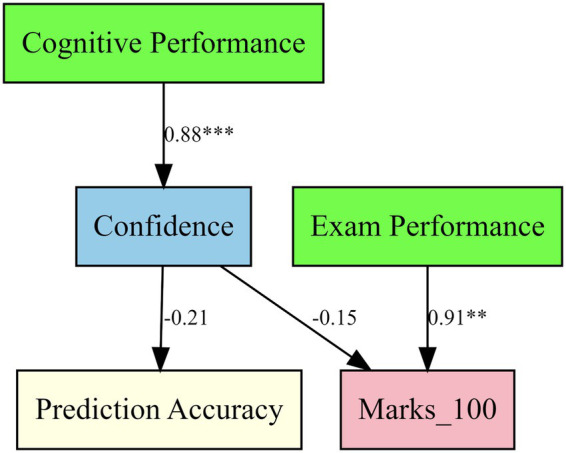
SEM path diagram.

A heatmap visualization was used to explain the multifaceted relationships between variables. This gradient color tool shows the strength and direction of each Pearson correlation. Correlations with strong positive values (e.g., between the exam scores and cognition) were dark green; weaker or negative correlations (e.g., gender and cognition) were lighter or red. This offers a fast visual overview of the interrelationship between metacognitive, behavioral, and demographic variables (see Figure 2).

One of the strengths of this work is that the research was conducted at two time points, which allowed one to visualize changes in confidence and metacognitive behavior prior to and after assessment. The fact that several indicators (prediction accuracy, confidence ratings, cognition scores) were included also made it possible to analyze the academic self-regulation in a multifaceted way.

Nevertheless, there are some constraints that must be noted. First, the questionnaire employed, even though it was reviewed by experts, was not validated, which restricted the generalizability. Second, the research was conducted in a single academic institution with a specific student cohort, which may limit the diversity and generalizability of the findings. Third, the cross-sectional design fails to consider long-term development or maintenance of metacognitive skills. Finally, the research was limited to midterm tests; the findings might not be applicable to other tests.

While the findings offer insights into students’ self-assessments and performance, it is important to note that these measures were self-reported. Consequently, the data may be subject to bias including social desirability bias, which limit the introspective accuracy. Students might provide responses that they believe are expected or socially desirable, and they may not always have complete insight into their own metacognitive processes. These factors could influence the accuracy of the self-assessment data. Nonetheless, self-report instruments were used here because metacognitive awareness is an internal process that is most feasibly assessed through introspective surveys in an educational setting. This approach is common in metacognitive research and was deemed appropriate for our exploratory study (Craig et al., 2020).

One explanation for this gender difference is a self-reported bias driven by gender socialization and confidence expression. Male students often exhibit higher academic self-efficacy and may overestimate or more readily report their use of metacognitive strategies, whereas female students tend to underestimate their abilities and thus report lower strategy use. This reflects a documented “male hubris, female humility” pattern in self-assessments, where men’s overconfidence and women’s modest self-evaluation emerge despite equivalent actual performance (Hofer et al., 2025). Such differences are partly rooted in societal norms For example, women may face social pressures to avoid appearing overconfident or “pretentious” leading to more restrained self-reports (Bodard et al., 2024). Consequently, the higher metacognitive strategy use reported by males in our study should be interpreted with caution because it likely reflects differences in self-perception rather than true disparities in metacognitive skill.

From a pedagogical perspective, these findings have important implications for teaching in hybrid learning environments and beyond. Instructors should address both the cognitive and emotional dimensions of student learning to enhance engagement and performance. A key strategy is to provide autonomy-supportive course structures; for example, incorporating pre-class digital modules and low-stakes practice quizzes can help students master foundational content at their own pace before high-stakes assessments. Such scaffolding helps build self-efficacy and confidence gradually, reducing anxiety. Additionally, instructors should offer timely, mastery-oriented feedback to strengthen students’ perceived control and lessen the emotional burden of poor performance. We also recommend integrating explicit self-monitoring activities into the curriculum—such as encouraging students to predict and reflect on their performance during flipped-classroom or case-based exercises. These “calibrated challenges” promote low-pressure practice in self-evaluation, helping normalize and improve metacognitive habits. Consistent with Lo’s (2023) framework, reducing grade-centric pressure and increasing student-led learning opportunities can improve the emotional climate. When framed through this cognitive–affective lens, such instructional approaches not only build metacognitive accuracy but also nurture student autonomy and emotional resilience.

## Conclusion

This research contributes to the small body of literature regarding the metacognitive awareness and confidence in pharmacy students. We confirm our assertions that metacognitive engagement, confidence calibration, and prediction accuracy are interrelated with academic performance. Gender can impact the way these processes are expressed, but more studies are required to explain this connection.

Based on these results, we suggest incorporating a metacognitive strategy training (e.g., self-explanation, prediction, peer feedback) into pharmacy curriculum. Moreover, reflective activities and tools to calibrate confidence can be useful to make students more accurate in their judgments. The teachers should also be sensitive to gender variations in metacognitive strategy application and confidence in the development of instructional interventions.

However, several limitations should be acknowledged. First and foremost, this work used a cross-sectional design that inherently limits the strength of our conclusions. It would also be valuable for future work to directly assess students’ emotional and motivational states alongside metacognitive measures. Specifically, we recommend incorporating brief, validated instruments—such as a course-specific self-efficacy scale or selected subscales from the Achievement Emotions Questionnaire (AEQ; Pekrun et al., 2011)—to capture perceived control and emotional regulation *in situ*. Short qualitative probes, such as brief reflective journal entries or post-exam open-ended questions, could also provide richer insight into the affective experiences that accompany metacognitive monitoring. Additionally, while we examined gender differences in metacognitive awareness and confidence, it is important to note that self-efficacy and engagement can vary across other subgroups in hybrid learning environments (Lo, 2023). Future research should examine whether such subgroup differences—including those related to prior academic preparation, learning preferences, or cultural background—account for variation in confidence calibration or prediction accuracy. All variables were measured at a single point in time, and thus we can only report associations among metacognitive confidence, self-evaluation, and performance; we cannot determine causal directionality. The lack of temporal data means that any interpretation of influence should be made with caution, and unmeasured factors (e.g., prior academic preparation or personality traits) might be contributing to the observed relationships. One notable limitation of this study is the absence of a qualitative component. The research relied solely on quantitative surveys and performance data without complementary qualitative evidence (such as student interviews or reflective journals). This lack of qualitative data precludes methodological triangulation, which might have strengthened the validity and richness of our conclusions. Triangulation—the corroboration of findings via multiple data sources—can yield a more comprehensive understanding of educational phenomena (Flick, 2004).

The study was also conducted in a single academic setting with a specific group of students, which may limit the generalizability of the findings. All measures of confidence and metacognitive judgments relied on self-reported instruments, introducing the possibility of response biases or inaccuracies in students’ self-perceptions. However, these tools are standard in metacognition research and were necessary to capture internal cognitive-affective states.

Future research should include multi-institution and cross-cultural samples to examine whether the observed relationships hold across diverse pharmacy curricula and educational contexts. Further studies are needed to review the development of metacognitive awareness in the long term and with various tests and groups of students. There should also be interventions that combine both cognitive and emotional areas like training the mindset and regulating anxiety. In summary, improved metacognitive support can lead to more self-managed, confident, and versatile pharmacy graduates that are ready for patient-centered care.

## Data Availability

The original contributions presented in the study are included in the article/Supplementary material, further inquiries can be directed to the corresponding author.

## Appendix: group statistics of midterm for gender.

**Table:**

Type of exam	Variable	Gender	*N*	Mean	Std. deviation	Std. error mean
Midterm exam-	Marks_100	Female	74	73.42	12.85	1.49
		Male	77	69.86	15.64	1.78
	Confidence score	Female	74	2.74	0.98	0.11
		Male	77	2.46	0.99	0.11
	Pred_before	Female	74	25.24	4.17	0.48
		Male	77	23.24	5.01	0.57
	Pred_After	Female	74	25.14	3.33	0.38
		Male	77	23.93	3.88	0.44
	Total score	Female	74	5.49	1.14	0.13
		Male	77	5.33	1.63	0.19
	Cognitive score	Female	74	22.29	5.34	0.62
		Male	77	20.14	5.73	0.65
