# Supplementary material for: Metacognitive awareness and confidence as predictors of academic performance in pharmacy students: insights from grade predictions and structural equation modeling
Source: Front Psychol. 2026 Feb 27;17:1720303. doi: 10.3389/fpsyg.2026.1720303 (PMC12982428; doi:10.3389/fpsyg.2026.1720303)
Supplement: Supplementary file 1 [file Table_1.docx]

| **Section** | **Item Code** | **Item Description** | **Response Scale** | **Scoring** |
| --- | --- | --- | --- | --- |
| Metacognitive Awareness | Q1-Q6 | How confident are you with your previous answer? | 1–5Likert (No idea→ Very confident) | Summed into Total_Cog |
| Confidence | Q7 | Overall, how confident are you in answering the questions? | 1–5 Likert (No idea→ Very confident) | Summed into Overall_Conf |
| Grade Prediction | Pred_before | Out of 20/30 marks, what do you expect your score in the coming midterm? | 0–100 numeric | Compared with actual score |
| Grade Prediction | Pred_after | Out of 20/30 marks, what do you expect your score in the coming midterm? | 0–100 numeric | Compared with actual score |

Supplementary Table S1. Survey Items, Response Scales, and Scoring Rules
